# Whole-genome sequencing and genetic diversity of severe fever with thrombocytopenia syndrome virus using multiplex PCR-based nanopore sequencing, Republic of Korea

**DOI:** 10.1371/journal.pntd.0010763

**Published:** 2022-09-12

**Authors:** Jingyeong Lee, Kyungmin Park, Jongwoo Kim, Seung-Ho Lee, Geum-Young Lee, Seungchan Cho, Heung-Chul Kim, Terry A. Klein, Jeong-Ah Kim, Jeewan Choi, Juwan Park, Dong-Hyun Song, Se Hun Gu, Hyeongseok Yun, Jung-Eun Kim, Daesang Lee, Gyeung Haeng Hur, Seong Tae Jeong, Il-Ung Hwang, Won-Keun Kim, Jin-Won Song

**Affiliations:** 1 Department of Microbiology, Korea University College of Medicine, Seoul, Republic of Korea; 2 BK21 Graduate Program, Department of Biomedical Sciences, Korea University College of Medicine, Seoul, Republic of Korea; 3 Chem-Bio Technology Center, Agency for Defense Development, Yuseong, Daejeon, Republic of Korea; 4 Force Health Protection and Preventive Medicine, Medical Department Activity-Korea/65th Medical Brigade, Unit 15281, United States of America; 5 Division of Emerging Infectious Diseases, Bureau of Infectious Diseases Diagnosis Control, Korea Disease Control and Prevention Agency, Cheongju, Republic of Korea; 6 Republic of Korea Armed Forces Medical Command, Seongnam, Republic of Korea; 7 The Fifth Preventive Medicine Unit of Republic of Korea Army, Pocheon, Republic of Korea; 8 Department of Orthopaedic Surgery, Sheikh Khalifa Specialty Hospital, Seoul National University Hospital, Seoul, Republic of Korea; 9 Department of Microbiology, College of Medicine, Hallym University, Chuncheon, Republic of Korea; 10 Institute of Medical Research, College of Medicine, Hallym University, Chuncheon, Republic of Korea; Kenya Agricultural and Livestock Research Organization, KENYA

## Abstract

**Background:**

Whole-genome sequencing plays a critical role in the genomic epidemiology intended to improve understanding the spread of emerging viruses. Dabie bandavirus, causing severe fever with thrombocytopenia syndrome (SFTS), is a zoonotic tick-borne virus that poses a significant public health threat. We aimed to evaluate a novel amplicon-based nanopore sequencing tool to obtain whole-genome sequences of Dabie bandavirus, also known as SFTS virus (SFTSV), and investigate the molecular prevalence in wild ticks, Republic of Korea (ROK).

**Principal findings:**

A total of 6,593 ticks were collected from Gyeonggi and Gangwon Provinces, ROK in 2019 and 2020. Quantitative polymerase chain reaction revealed the presence of SFSTV RNA in three *Haemaphysalis longicornis* ticks. Two SFTSV strains were isolated from *H*. *longicornis* captured from Pocheon and Cheorwon. Multiplex polymerase chain reaction-based nanopore sequencing provided nearly full-length tripartite genome sequences of SFTSV within one hour running. Phylogenetic and reassortment analyses were performed to infer evolutionary relationships among SFTSVs. Phylogenetic analysis grouped SFTSV Hl19-31-4 and Hl19-31-13 from Pocheon with sub-genotype B-1 in all segments. SFTSV Hl20-8 was found to be a genomic organization compatible with B-1 (for L segment) and B-2 (for M and S segments) sub-genotypes, indicating a natural reassortment between sub-genotypes.

**Conclusion/Significance:**

Amplicon-based next-generation sequencing is a robust tool for whole-genome sequencing of SFTSV using the nanopore platform. The molecular prevalence and geographical distribution of SFTSV enhanced the phylogeographic map at high resolution for sophisticated prevention of emerging SFTS in endemic areas. Our findings provide important insights into the rapid whole-genome sequencing and genetic diversity for the genome-based diagnosis of SFTSV in the endemic outbreak.

## Author summary

Next-generation sequencing (NGS) is important in understanding the genetic diversity, genomic epidemiology, and transmission chain of emerging viruses of concern. Severe fever with thrombocytopenia syndrome (SFTS) is a zoonotic vector-borne infectious disease with a high mortality rate. Approximately 170 SFTS cases are reported annually in Republic of Korea. We assessed a multiplex polymerase chain reaction-based NGS method for whole-genome sequencing of SFTS virus (SFTSV) and monitored the prevalence of SFTSV in wild ticks. We developed an amplicon-based NGS method to obtain whole-genome sequences of SFTSV using the nanopore sequencing. A total of 6,593 ticks were collected from Gyeonggi and Gangwon Provinces in 2019 and 2020. The infection and minimum infection rates of SFSTV were 0.34% and 0.05%, respectively. We recovered whole-genome sequences of SFTSV from three *Haemaphysalis longicornis* ticks collected from Pocheon and Cheorwon, enhancing the high-resolution phylogeographical analysis for potential prevention of SFTS outbreaks. Phylogenetic analysis showed that SFTSV Hl20-8 from Cheorwon was a reassortant compatible with the B-1 and B-2 sub-genotypes. Our findings provide important insights into the target enrichment-based NGS and genetic diversity for the genome-based diagnosis of SFTSV in the endemic outbreak.

## Introduction

Severe fever with thrombocytopenia syndrome (SFTS) is a zoonotic vector-borne infectious disease with clinical symptoms including acute fever above 38°C, thrombocytopenia, leukopenia, and multiple organ dysfunction [1]. SFTS was first reported in China, followed by several reports from many other countries including Republic of Korea (ROK), Japan, Vietnam, and Taiwan [2–6]. Although the mean case mortality rate has varied among countries and with time, the average fatality rate of SFTS has remained relatively high in China (5.3–16.2%), ROK (23.3%), and Japan (27%) [7–9]. According to Korea Disease Control and Prevention Agency, approximately 170 SFTS patients have been recorded annually in the ROK [10]. There are no effective vaccines and antiviral therapeutics for SFTS despite the significant morbidity and mortality of the disease.

Dabie bandavirus (formerly called SFTS virus, SFTSV) is an enveloped negative-sense single-stranded RNA virus, belongs to the family *Phenuiviridae*, order *Bunyavirales*, [11]. The SFTSV genome contains three segments, including the large (L) segment, which encodes an RNA-dependent RNA polymerase; a medium (M) segment, which encodes two surface glycoproteins Gn and Gc; a small (S) segment, which encodes a nucleoprotein (NP) and a nonstructural S segment (NS) protein, respectively [12]. The life cycle and transmission mechanisms of SFTSV in nature remain unclear, although transmission via arthropod vectors is considered the most credible route, similar to that for other members of *Phenuiviridae* [13]. The Asian longhorned tick (*Haemaphysalis longicornis*) is the main transmission vector of SFTSV in East Asian countries [14]. SFTSV RNA has also been detected in several other tick species, including *H*. *flava*, *Rhipicephalus microplus*, *Amblyomma testudinarium*, *Dermacentor nuttalli*, *Hyalomma asiaticum*, and *Ixodes nipponensis* in endemic areas [15–18]. Furthermore, human-to-human transmission by contact with blood or body fluid from SFTS patients has been reported in ROK and China [19,20].

Next-generation sequencing (NGS) plays a critical role in understanding the genetic diversity, molecular epidemiology, and transmission chain of virus outbreaks [21–23]. Several NGS approaches, including sequence-independent single-primer amplification, small RNA deep sequencing, and target enrichment methods have been utilized for whole-genome sequencing (WGS) of emerging viruses with low viral copy numbers [24–27]. The MinION system (Oxford Nanopore Technologies, London, UK) is a portable device for real-time sequencing in field situations or hospitals [28,29]. Multiplex polymerase chain reaction (PCR)-based nanopore sequencing has been used to obtain nearly complete genome sequences of Hantaan virus from natural reservoir hosts to define the phylogeographical association and molecular evolution in the ROK [30]. Amplicon-based NGS methods with a nanopore system were developed to acquire full-length genomic sequences of severe acute respiratory syndrome coronavirus 2 for phylogenetic and epidemiological analyses [31]. However, to our knowledge, the amplicon-based NGS is yet to be performed for complete genome sequencing of SFTSV.

## Methods

### Ethics statement

This study was approved by the Korea University Institutional Animal Care and Use Committee (KU-IACUC) and performed with strict accordance to the recommendations of the KU-IACUC (No. #2019–171) guideline. All experiments were conducted in an animal biosafety level 3 (ABSL3) laboratory at Korea University.

### Tick collection and species identification

**Fig 1:**
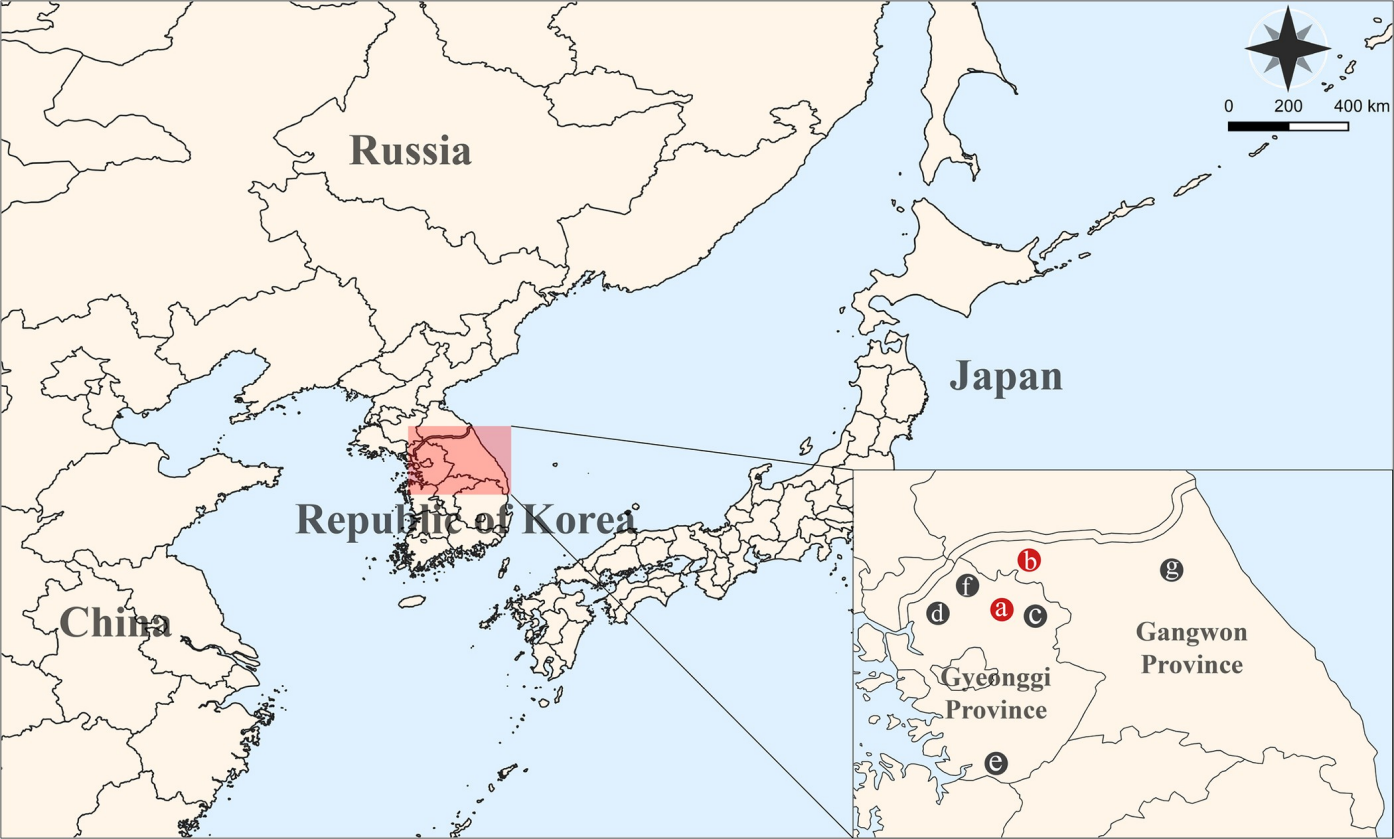
Geographical locations of tick collection in Gyeonggi and Gangwon Provinces, Republic of Korea, during 2019–2020. The red circles indicate the severe fever with thrombocytopenia syndrome virus (SFTSV) RNA positive sites: a, Pocheon in Gyeonggi Province; b, Cheorwon in Gangwon Province. The black circles show the regions where no SFTSV RNA was detected: c, Gapyeong; d, Paju; e, Pyeongtaek; and f, Yeoncheon in Gyeonggi Province; g, Inje in Gangwon Province. The geographic map was created by a Quantum Geographical Information System 3.10 software for Mac and modified using Adobe Illustrator CC 2019.

Tick sampling (n = 6,593) was performed in Gyeonggi and Gangwon Provinces, ROK, during 2019–2020 (Fig 1). Ticks were collected by well-trained researchers (US Forces Korea and the ROKA) from military bases located in seven areas: Gyeonggi (Gapyeong, Paju, Pocheon, Pyeongtaek, and Yeoncheon) and Gangwon (Cheorwon and Inje) Provinces. Ticks were captured using the dragging method described in a previous study [32]. All samples were identified by morphological characterization and life stages, and subsequently transported to ABSL3 facility at Korea University [33,34]. The captured ticks were identified as *H*. *longicornis* (n = 5,607, 85.04%), *H*. *flava* (n = 309, 4.69%), *H*. *phasiana* (n = 1, 0.02%), *I*. *nipponensis* (n = 666, 10.10%), and *I*. *persulcatus* (n = 10, 0.15%) (Table 1). Among the life cycle stages of ticks, nymphs accounted for most of all ticks (n = 5,709, 86.59%), followed by larvae (n = 407, 6.17%), males (n = 264, 4.00%), and females (n = 213, 3.23%) (Table 2).

**Table 1:** Geographical distribution of tick species collected from Gyeonggi and Gangwon Provinces, Republic of Korea, during 2019–2020.

Province	Region	Number of ticks collected					
		*Haemaphysalis longicornis*	*H*. *flava*	*H*. *phasiana*	*Ixodes nipponensis*	*I*. *persulcatus*	Total (%)
Gyeonggi	Gapyeong	938	13	0	2	0	953 (14.45)
	Paju	945	8	0	1	0	954 (14.47)
	Pocheon	906	25	0	22	0	953 (14.45)
	Pyeongtaek	2,156	260	1	591	0	3,008 (45.62)
	Yeoncheon	427	1	0	37	0	465 (7.05)
Gangwon	Cheorwon	235	2	0	0	0	237 (3.59)
	Inje	0	0	0	13	10	23 (0.35)
**Total (%)**		**5,607 (85.04)**	**309 (4.69)**	**1 (0.02)**	**666 (10.10)**	**10 (0.15)**	**6,593 (100)**

**Table 2:** Total number of tick species based on life cycle stages.

Year	Species	Life cycle stage				Total (%)
		Larva(e)	Nymph(s)	Adult (Male)	Adult (Female)	
2019	*Haemaphysalis longicornis*	0	5,136	6	31	5,173 (78.46)
	*H*. *flava*	0	244	32	28	304 (4.61)
	*H*. *phasiana*	0	1	0	0	1 (0.02)
	*Ixodes nipponensis*	231	66	219	150	666 (10.10)
	*I*. *persulcatus*	0	2	6	2	10 (0.15)
2020	*H*. *longicornis*	176	255	1	2	434 (6.58)
	*H*. *flava*	0	5	0	0	5 (0.08)
**Total (%)**		**407 (6.17)**	**5,709 (86.59)**	**264 (4.00)**	**213 (3.23)**	**6,593 (100)**

### RNA extraction and cDNA synthesis

Tick specimens (larvae and nymphs, n = 1–30; adult, n = 1) were homogenized with stainless steel beads in 700 μL phosphate buffered saline using a FastPrep-24 5G benchtop homogenizer (MP Biomedicals, Irvine, CA, USA). Homogenates were centrifuged for 5 min at 8,600 × g and 4°C. The recovered supernatants were stored at −80°C until use. Total RNA was extracted from 250 μL aliquots of the supernatants using TRIzol LS Reagent (Ambion, Austin, TX, USA) according to the manufacturer’s instructions. cDNA was synthesized from 1 μg of total RNA using a High Capacity RNA-to-cDNA kit (Applied Biosystems, Foster City, CA, USA) with SFTSV-PHS (5′-ACA CAR AGA CSS CC-3′).

### Quantitative polymerase chain reaction (qPCR)

qPCR was performed from the prepared cDNA using SYBR Green PCR Master Mix (Applied Biosystems) on a Quantstudio 5 Flex Real-Time PCR System (Applied Biosystems). The reaction mixture contained 5 μL of 2X SYBR Green PCR Master Mix, 1 μL of 5 μM each primer (final concentration, 0.5 μM), 3 μL of nuclease-free water, and 1 μL cDNA template in a total volume of 10 μL. The forward and reverse primer sequences are SFTS-L-F (5’-ACC TCT TTG ACC CTG AGT TWG ACA-3’) and SFTS-L-R (5’-CTG AAG GAG ACA GGT GGA GAT GA-3’), respectively. The cycling conditions consisted of denaturation at 95°C for 10 min, followed by 45 cycles at 95°C for 15 s and 60°C for 1 min. The cutoff C_t_ value was 40 in the assay.

### Reverse transcription-polymerase chain reaction (RT-PCR)

RT-PCR was performed from total RNA of inoculated supernatants and cells to validate the isolation of SFTSV. Total RNA was extracted using TRIzol LS Reagent (Ambion) according to the manufacturer’s instructions. cDNA was synthesized from 1 μg of total RNA using the High Capacity RNA-to-cDNA kit (Applied Biosystems) with SFTSV-PHS. The reaction mixture contained 2.5 μL of 10X reaction buffer (JMR Holdings, London, UK), 200 μM dNTP (Elpis Biotech, Daejeon, Korea), 0.25 U of SuperTherm Taq polymerase (JMR Holdings), 10 μM each primer, and 1.5 μL of cDNA template in a total volume of 25 μL. SFTSV-specific primer sequences were SFTSV-L1F (outer and inner): 5’-ACA CAG AGA CGC CCA GAT-3’, SFTSV-L343R (outer): 5’-CAT CCC ATC AGA ACC ATC AT-3’, and SFTSV-L263R (inner): 5’-TGA AGT CAT GAT TGA TCT-3’ for the L segment [35]; SFTSV-M482F (outer): 5’-TCT GCA GTT CAG ACT CAG GGA-3’, SFTSV-M1242R (outer): 5’-GAC GTG TAT TGC TGT TTT CCC-3’, SFTSV-M549F (inner): 5’-TGT TGC TTG TCA GCC TAT GAC-3’, and SFTSV-M1222R (inner): 5’-CAA CCA ATG ATC CTG AGT GGA-3’ for the M segment [12]; SFTSV-S1123F (outer): 5’- TAA ACT TCT GTC TTG CTG GCT CC-3’, SFTSV-S1589R (outer and inner): 5’-ATC AAG AAG CTG AAG GAG ACA G-3’, and SFTSV-S1169F (inner): 5’-TGG TGA AGG CAT CTT GCC A-3’for the S segment [36]. The cycling conditions for first and nested PCR consisted of initial denaturation at 95°C for 5 min, followed by 6 cycles of denaturation at 95°C for 40 s, annealing at 37°C for 40 s, elongation at 72°C for 1 min, followed by 32 cycles of denaturation at 95°C for 40 s, annealing at 42°C for 40 s, elongation at 72°C for 1 min and then final elongation at 72°C for 7 min using ProFlex PCR System (Life Technology, Carlsbad, USA).

### Cell lines

Vero E6 cells (ATCC, #DR-L2785) were purchased from ATCC. The cell lines were seeded and maintained in Dulbecco’s modified Eagle’s medium (DMEM) supplemented with 10% fetal bovine serum (FBS), 1% HEPES buffer, 1% L-glutamine (Lonza, Basel, Switzerland), and 0.1% gentamicin (Gibco, Life Technologies, Carlsbad, CA, USA). The plates were incubated at 37°C with 5% CO_2_ in an incubator.

### Virus isolation

The aliquots of SFTSV-positive supernatant were inoculated into the prepared Vero E6 cells. After 90 min of adsorption, the excess inoculum was discarded, and the viral suspension was replaced with 5.5 mL of DMEM containing 5% FBS, 1% HEPES buffer, 1% L-glutamine, and 0.1% gentamicin. The cells were incubated at 37°C with 5% CO_2_ in an incubator and passaged at 7–10 days intervals.

### Plaque assay

2×10^6^ Vero E6 cells were seeded in the each well of 6-well plates. After overnight incubation at 37°C with 5% CO_2_, the each well was washed twice with PBS and inoculated with 10-fold serially diluted SFTSV. After 90 min absorption at 37°C, the cells were overlaid with the overlay medium and medium-melting-point agarose mix (2:1 ratio). The plaques were incubated at 37°C for 5 days and then visualized by staining the cells with 5% neutral red solution (Sigma-Aldrich, Burlington, USA).

### Multiplex PCR

Multiplex PCR primers were designed for WGS of SFTSV L, M, and S segments. cDNA was amplified using SFTSV-specific primer mixtures and Solg 2X Uh-Taq PCR Smart mix (Solgent, Daejeon, ROK) according to the manufacturer’s instructions. The composition of 25 μL of the reaction mixture was 12.5 μL of 2× Uh pre-mix, 1.0 μL cDNA template, 10.0 μL of 0.5 μM each primer mixture (final concentration, 0.2 μM), and 1.5 μL of distilled water. The first PCR cycling was performed with the following cycling conditions: initial denaturation at 95°C for 15 min, 40 cycles at 95°C for 20 s, 50°C for 40 s, 72°C for 1 min, and final elongation at 72°C for 3 min. The second PCR was conducted in a 25 μL reaction mixture containing 12.5 μL 2× Uh pre-mix, 1.0 μL of the first PCR product, 10.0 μL of 0.5 μM each primer mixture (final concentration, 0.2 μM), and 1.5 μL of distilled water. The cycling conditions included an initial denaturation at 95°C for 15 min, followed by 25 cycles at 95°C for 20 s, 50°C for 40 s, 72°C for 1 min, and final elongation at 72°C for 3 min. The primer sequences are shown in S1 Table.

### Nanopore sequencing

The DNA library was prepared using a Ligation Sequencing Kit (SQK-LSK109) with a Native Barcoding Kit (EXP-NBD104; Oxford Nanopore Technologies) according to the manufacturer’s instructions. The libraries were barcoded, pooled, and ligated to sequencing adapters. Purified libraries were loaded onto FLO-MIN106 (R9.4; Oxford Nanopore Technologies) and sequenced using the MinION device (Oxford Nanopore Technologies). Basecalling was performed by Guppy (v3.0.3) embedded in the MinIT system (Oxford Nanopore Technologies). Raw data were demultiplexed and the adaptor sequences were trimmed using MinKNOW software (Oxford Nanopore Technologies). The filtered reads were assembled into a single file using Porechop v.9.0. Viral reads were mapped to the reference genome sequences of SFTSV SPL114A, and consensus sequences were extracted by CLC Genomics Workbench (v7.5.2; Qiagen, Hilden, Germany). Manual polishing was performed using the indel error-correction method described previously [30].

### Illumina sequencing

DNA libraries were prepared using TruSeq Nano DNA low-throughput sample preparation kit (Illumina, San Diego, CA, USA) according to the manufacturer’s instructions. DNA templates were mechanically sheared by an M220 focused ultrasonicator (Covaris, Woburn, MA, USA). The fragmented amplicons were size-selected, A-tailed, ligated with indexes and adapters, and enriched by PCR. The quality and concentration of libraries were evaluated using the DNA 1000 chip kit (Agilent Technologies, Santa Clara, CA, USA) on a bioanalyzer (Agilent Technologies). The libraries were quantified by qPCR using Library Quantification Kit (KAPA Biosystems, Wilmington, MA, USA) on a Quantstudio 5 Flex Real-Time PCR System (Applied Biosystems). The pooled libraries were sequenced on a MiSeq benchtop sequencer (Illumina) with 2 × 150 bp using a MiSeq reagent kit v2 (Illumina). Adapter and index sequences were trimmed from the raw data and filtered reads were mapped to the reference genomic sequences of SFTSV SPL114A by CLC Genomics Workbench (v7.5.2; Qiagen). The consensus sequences were extracted from analyzed viral reads.

### Rapid amplification of cDNA ends (RACE)

PCR was performed using 3′ and 5′ RACE System for Rapid Amplification of cDNA Ends version 2.0 (Invitrogen, Carlsbad, CA, USA), according to the manufacturer’s instructions. The primer sequences are shown in S2 Table.

### Phylogenetic analysis

The tripartite genomic sequences of SFTSV were aligned using the Clustal W method in Lasergene version 5 (DNASTAR, Madison, WI, USA). Phylogenies were generated using the best fit GTR+G+I (for all segments) substitution models of evolution by the maximum likelihood method in MEGA7 [37]. The topologies were assessed by bootstrap analysis for 1,000 iterations.

### Genetic reassortment analysis

Graph incompatibility-based reassortment finder (GiRaF) analysis was performed to estimate genetic reassortment events [38]. Alignments of SFTSV tripartite genomes were used as an input source for Bayesian analysis [39]. The optimal evolutionary models were determined using MEGA7. A total of 1,000 unrooted candidate trees were generated using GTR+G+I substitution model sampled every 200 iterations with a 25% burn-in. The analysis was repeated ten times with each independent MrBayes-based tree data. The default value of a confidence threshold was 0.7 for the data set.

## Results

### Epidemiological surveillance of SFTSV

Tick samples were pooled by criteria of the life cycle stage. If larvae and nymphs were captured at identical areas, they were pooled up to 30 individuals. A total of three pooled tick samples (Hl19-31-4, Hl19-31-13, and Hl20-8) were found to harbor SFTSV RNA. SFTSV Hl19-31-4 and Hl19-31-13 were collected from Pocheon in Gyeonggi Province, in 2019. SFTSV Hl20-8 was identified from Cheorwon in Gangwon Province, in 2020. Infection and minimum infection rates were 0.34% (3/886) and 0.05% (3/6,582), respectively (Table 3).

**Table 3:** Molecular prevalence of severe fever with thrombocytopenia syndrome virus (SFTSV) from ticks collected from Gyeonggi and Gangwon Provinces, Republic of Korea, during 2019–2020.

Year	Species	No. of ticks ^a^	No. of tick pools ^b^	SFTSV RNA positivity ^c^	
				IR (%) ^d^	MIR (%) ^e^
2019	*Haemaphysalis longicornis*	5,173	317	2/317 (0.63)	2/5,173 (0.04)
	*H*. *flava*	304	109	0/109	0/304
	*Ixodes nipponesis*	666	421	0/421	0/666
2020	*H*. *longicornis*	434	37	1/37 (2.70)	1/434 (0.23)
	*H*. *flava*	5	2	0/2	0/5
**Total**		**6,582**	**886**	**3/886 (0.34)**	**3/6,582 (0.05)**

^a^ A total of 11 ticks were not investigated in this study.

^b^ Criteria of pooling number: Adult (Male/Female) 1, Nymph 1–30, Larva 1–30; ticks captured in different sites were not pooled.

^c^ SFTSV RNA positivity was determined using SFTSV-specific quantitative polymerase chain reaction.

^d^ IR: infection rate.

^e^ MIR: minimum infection rate.

### Virus isolation of SFTSV

Partial genomic sequences of SFTSV were identified from inoculated supernatants and cells (S1 Fig). The first plaques of SFTSV Hl19-31-4 and Hl20-8 were confirmed at 5 days post-inoculation, and the number of infectious particles was 1.2×10^6^ and 2.0×10^6^ PFU/mL, respectively (S2 Fig).

### Multiplex PCR-based NGS of SFTSV using nanopore and Illumina sequencing

**Fig 2:**
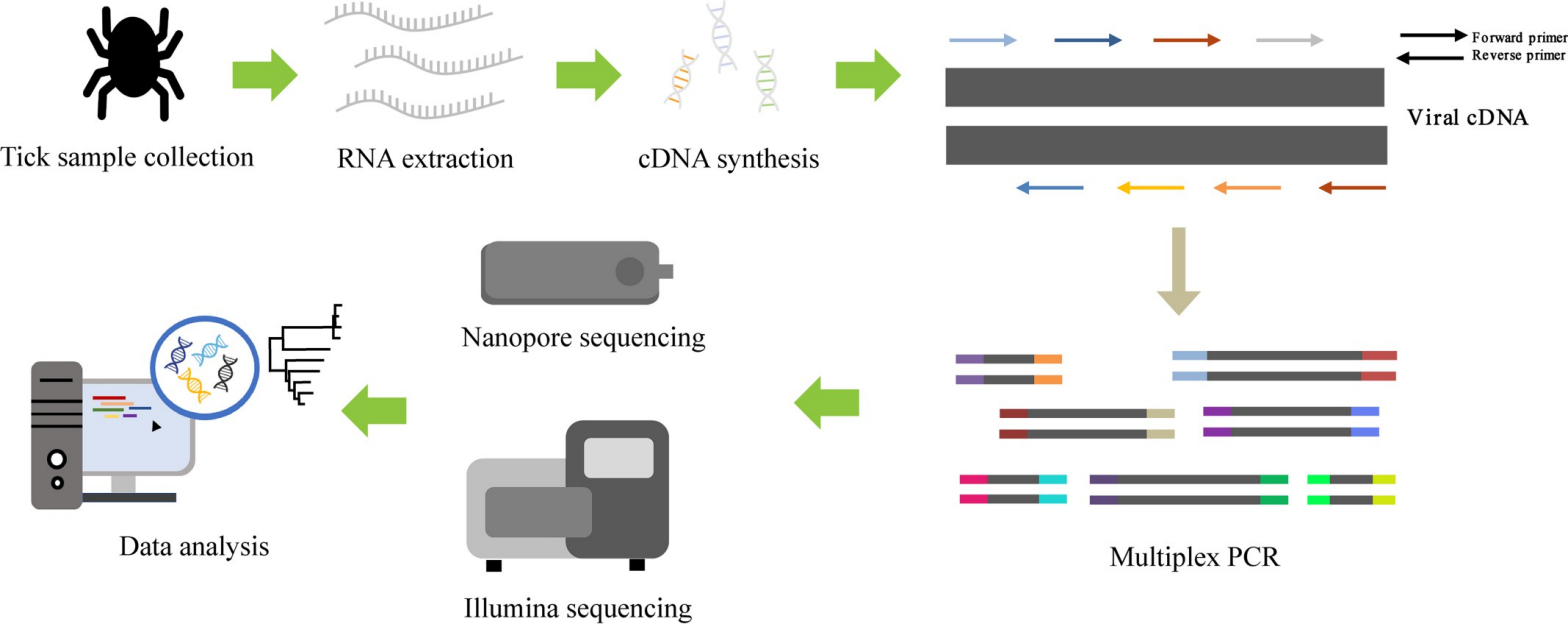
Workflow overview of multiplex PCR-based next-generation sequencing for whole-genome sequencing of severe fever with thrombocytopenia syndrome virus (SFTSV). Total RNA was extracted from tick samples and cDNA was synthesized with SFTSV-specific oligonucleotide primer (SFTSV-PHS). cDNA was enriched using designed SFTSV-specific primer mixtures for the next-generation sequencing. Amplified libraries were pooled, ligated to sequencing adapter, and sequenced using nanopore and Illumina sequencing according to each manufacturer’s instructions. Raw data were filtered and analyzed by CLC Genomics Workbench (v7.5.2).

**Fig 3:**
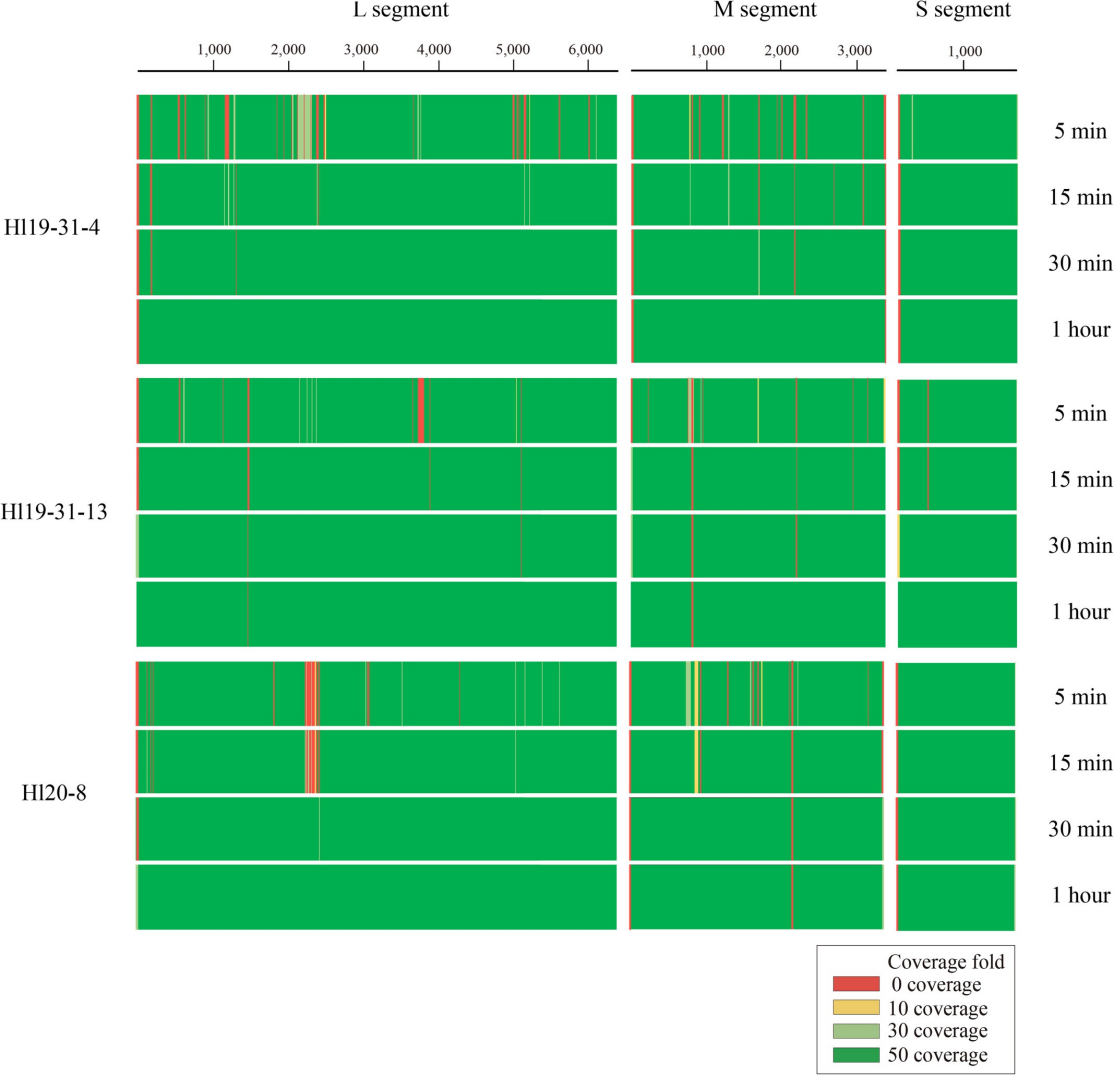
Average depth of coverages of severe fever with thrombocytopenia syndrome virus (SFTSV) L, M, and S segments using multiplex PCR-based nanopore sequencing at different cumulative running times. The average depth of coverages of SFTSV tripartite genomes was shown by using amplicon-based nanopore sequencing at 5 min, 15 min, 30 min, and 1 h. The colors represent the coverage fold; 0 coverage (red), from 1 to 10 coverage (yellow), from 11 to 30 coverage (light green), from 31 to 50 and over 50 (green).

The workflow overview of multiplex PCR-based NGS for whole-genome sequencing of SFTSV is shown in Fig 2. Using multiplex PCR-based nanopore sequencing, nearly whole-genome sequences of SFTSV were recovered from the collected tick samples collected from Gyeonggi and Gangwon Provinces, ROK. The mean genome coverages were 99.43% for the L segment, 99.47% for the M segment, and 99.20% for the S segment, with over 50× depth of coverage at all regions for each segment after one hour of nanopore sequencing (Fig 3 and S3 Table). Average viral reads and depth for SFTSV tripartite genomes were determined using the mapped reads corresponding to sequencing running times.

The tick specimens were sequenced using the Illumina MiSeq system to obtain whole-genome sequences of SFTSV. The coverage rates of SFTSV were 99.43% for the L segment, 99.47% for the M segment, and 99.20% for the S segment (S4 Table). The 3′ and 5′ terminal sequences of SFTSV L, M, and S segments were determined using RACE PCR.

### Genetic diversity and genome exchange of SFTSV using the phylogenetic inference

**Fig 4:**
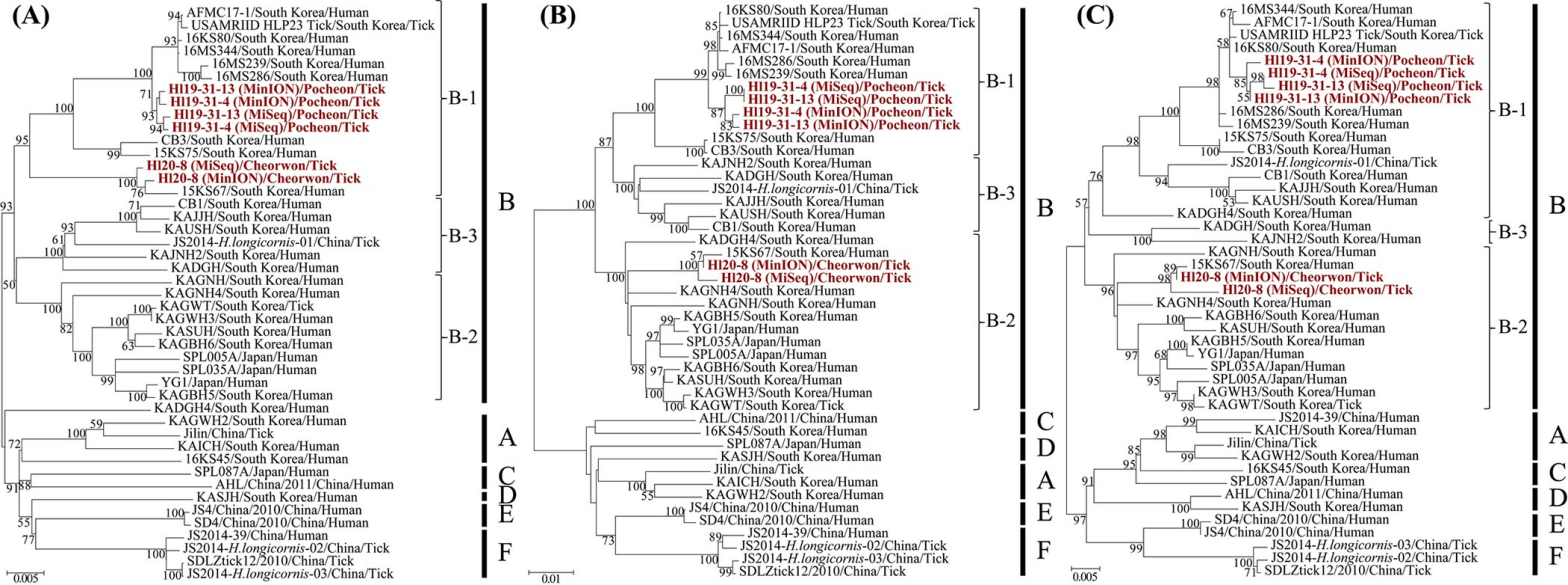
Phylogenetic analyses inferred to open reading frame (ORF) sequences of severe fever with thrombocytopenia syndrome virus (SFTSV) tripartite genomes. The phylogenetic trees were generated using maximum likelihood methods in MEGA7 with bootstrap 1,000 iterations based on the complete ORF regions of the SFTSV (A) L (17–6,270 nt), (B) M (19–3,240 nt), and (C) S (29–1,702 nt) segments. The scale bars indicate the number of nucleotide substitutions per site. The numbers at each node are bootstrap probabilities determined for 1,000 replicates. The SFTSV obtained in this study is shown in red lettering. The genetic clades indicate six genotypes (A-F) and three sub-genotypes (B-1 to B-3) in the right panel. The genomic sequences of SFTSV in this figure are described in S5 Table.

The phylogenetic analysis showed that Hl19-31-4 and Hl19-31-13 from Pocheon were clustered with sub-genotype B-1 in all segments of SFTSV (Fig 4). The phylogenetic patterns of SFTSV L, M, and S segments demonstrated that Hl20-8 from Cheorwon had differing levels of incongruence in the phylogenies. The L segment of SFTSV Hl20-8 shared common ancestors with sub-genotype B-1, whereas M and S segments formed a clade with sub-genotype B-2.

**Fig 5:**
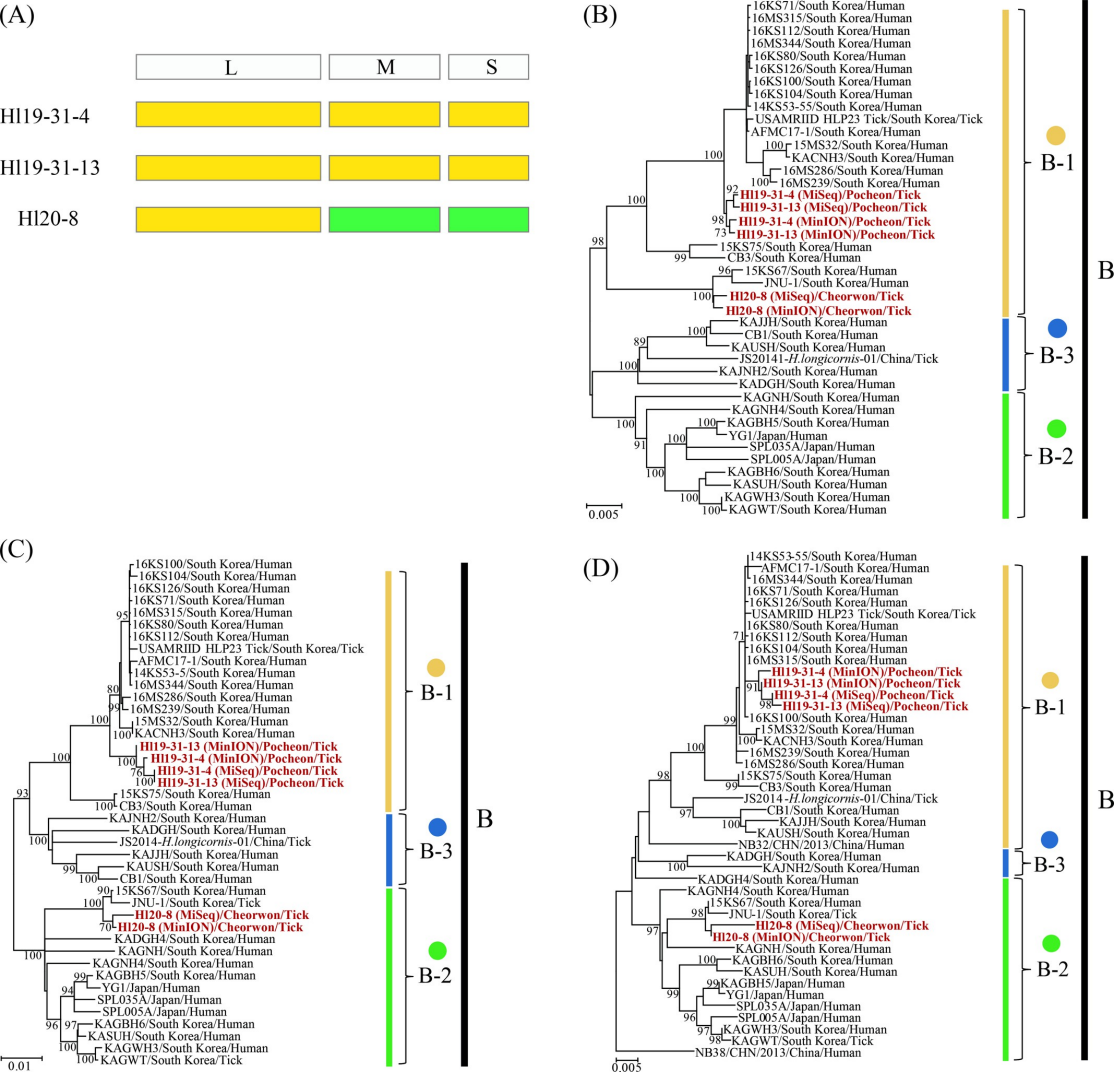
Genetic reassortment of severe fever with thrombocytopenia syndrome virus (SFTSV) Hl20-8 from Cheorwon, Republic of Korea. (A) The tripartite genomes of SFTSV were described using a pairwise distance model estimated using the graph incompatibility-based reassortment finder software. Phylogenetic trees were generated using the maximum likelihood method in MEGA7 with 1,000 bootstrap iterations based on the ORFs of the SFTSV (B) L (17–6,270 nt), (C) M (19–3,240 nt), and (D) S (29–1,702 nt) segments. SFTSVs are color-coded according to sub-genotype classification as follows: yellow, B-1; green, B-2; blue, B-3. Newly recovered SFTSV genomes are shown in red. The genomic sequences of SFTSV in this figure are described in S5 Table.

The occurrence of genetic reassortment of SFTSV was estimated using the GiRaF software (Fig 5). The genome exchanges were detected in SFTSV Hl20-8 between B-1 and B-2 sub-genotypes with over 0.9 confidence levels. The genome composition of SFTSV Hl20-8 was compatible with the B-1 sub-genotype (for the L segment) and the B-2 sub-genotype (for the M and S segments).

## Discussion

High-throughput sequencing technologies have become robust tools for improving approaches to point-of-care diagnostics and molecular epidemiology of emerging diseases when used to track sources of epidemic infections [40–42]. NGS-based genomic surveillance with MinION system has been applied to elucidate virus characterization and transmission dynamics of Ebola virus, Zika virus, and severe acute respiratory syndrome coronavirus 2 in the field [43–45]. The extension of SFTSV genome sequences has been limited by the lack of a relevant sequencing assay with designed primers for attaining complete genomic sequences. We established a multiplex PCR-based NGS that generated the full-length genomic sequence of SFTSV from ticks using the primer set specific for SFTSV L, M, and S segments. The multiplex primers were designed from reference sequences deposited in NCBI, enabling whole-genome sequencing of all genotypes of SFTSV. Given the genetic diversity among variants of SFTSV, further studies are needed to determine the sensitivity of the assay on clinical and tick samples containing varied genotypes and ultra-low viral RNA copy numbers.

The spread of SFTSV is attributed to the geographical distribution of arthropod vectors and contacts between humans and infected tick populations [13]. Approximately 170 SFTS cases occur annually in ROK, affecting both military personnel and civilians [10]. Yoo et al. described the epidemiological association between molecular prevalence of SFTSV and tick populations with ecological environments in endemic areas [46]. Epidemiological surveillance has been performed to clarify the serological and molecular prevalence of SFTSV and the distribution of their reservoirs in the ROK [47–49]. Phylogeographic analysis of SFTSV elucidated an epidemiological association between viral sequences from ROK Army soldier with SFTS and wild ticks collected from the putative infection site [35]. The additional genome sequences of SFTSV with geographical information enable to define the phylogenetic and spatial relationships of SFTS patients with infectious sources at high-resolution [50,51]. In this study, whole-genome sequences of SFTSV were newly obtained from ticks (*H*. *longicornis*) collected from Pocheon and Cheorwon, enhancing the resolution of a phylogeographic map for the sophisticated prevention of SFTSV infections in ROK. Our findings demonstrated the development of a high-resolution phylogeographical database of SFTSV for mitigating SFTS outbreaks to humans in the endemic areas.

Genetic reassortment confers a capacity to generate novel variants by which segmented RNA viruses shuffle the viral genomes [52]. Genetic reassortment events promote alterations in viral characteristics including host immunity evasion, transmissibility, and virulence to humans [53,54]. Genome exchanges occur among intra- and inter-lineage of SFTSV in nature, resulting in the emergence of new genotypes [55]. Phylogenetic clustering patterns revealed the six pure genotypes (A-F) and nine reassortments (R1–R9) with different rates of mortality, supporting the genotype-dependent pathogenic potential of SFTSV [9,56]. The distinct distribution of SFTSV genotypes may be correlated with varied mortality rates in China (5.3–16.2%), ROK (23.3%), and Japan (27%) [2,7–9]. The majority of Korean SFTSV belonged to genotype B (69.2%), including three different sub-genotypes; the most dominant sub-lineage was B-2 (36.1%), followed by B-3 (21.1%) and B-1 (12%) [9]. In this study, the phylogenies of SFTSV Hl20-8 from Cheorwon demonstrated incongruent phylogenetic patterns of the tripartite genomes, indicating the differential evolution of each segment. These results suggest that SFTSV Hl20-8 formed a genomic organization compatible with the B-1 sub-genotype (for the L segment) and the B-2 sub-genotype (for the M and S segments). To better understand the pathogenicity and evolutionary complexity, further studies should conduct continuous collection, epidemiological surveys, and risk assessment of various SFTSV genotypes, ROK.

In conclusion, we developed the multiplex PCR-based NGS for SFTSV from tick samples using nanopore sequencing. The entire genomic sequences of SFTSV were newly recovered from ticks (*H*. *longicornis*) collected from Pocheon and Cheorwon, supporting the phylogeographical analysis at high resolution for sophisticated prevention of SFTS outbreaks in ROK. Phylogenetic and reassortment analyses demonstrated that SFTSV Hl20-8 from Cheorwon is a reassortant compatible with B-1 and B-2 sub-genotypes. These results provide important insights into the amplicon-based NGS and genetic diversity for the rapid genome-based diagnosis of SFTSV in endemic outbreaks.

## Supporting information

S1 Fig Reverse-transcription polymerase chain reaction (RT-PCR) to validate isolation of severe fever with thrombocytopenia syndrome virus (SFTSV). RT-PCR was continually performed to confirm isolation of SFTSV (A) Hl19-31-4 and (B) Hl20-8 strains from supernatants and cells from infected Vero E6 cells.
(TIF)

S2 Fig The plaque assay of severe fever with thrombocytopenia syndrome virus (SFTSV) Hl19-31-4 and Hl20-8 strains. SFTSV Hl19-31-4 and Hl20-8 strains were quantified on 6-well plates with Vero E6 monolayers. Each well indicates different dilutions from top-left to right: no dilution, dilutions at 1:10^1^, and 1:10^2^, and bottom-left to right: dilutions at 1:10^3^, 1:10^4^, and 1:10^5^, respectively. The first plaques of SFTSV (A) Hl19-31-4 and (B) Hl20-8 were confirmed at 5 days post-inoculation, and the number of infectious particles was 1.2×10^6^ and 2.0×10^6^ PFU/mL, respectively.
(TIF)

S1 Table Multiplex polymerase chain reaction primer information for severe fever with thrombocytopenia syndrome virus L, M, and S segments. (PDF)

S2 Table Oligonucleotide primers to obtain 3’and 5’ends genomic sequences of L, M and S segments of severe fever with thrombocytopenia syndrome virus using rapid amplification of cDNA ends polymerase chain reaction in this study. (PDF)

S3 Table Summary of mapped reads and average depth of multiplex polymerase chain reaction-based nanopore sequencing of severe fever with thrombocytopenia syndrome virus. (PDF)

S4 Table Mapped reads and average depth of multiplex polymerase chain reaction-based Illumina sequencing of severe fever with thrombocytopenia syndrome virus. (PDF)

S5 Table Accession numbers of genomic sequences of severe fever with thrombocytopenia syndrome virus used in this study. (PDF)
