# Supplementary figures and images for: Whole-genome sequencing and genetic diversity of severe fever with thrombocytopenia syndrome virus using multiplex PCR-based nanopore sequencing, Republic of Korea
Source: PLoS Negl Trop Dis. 2022 Sep 12;16(9):e0010763. doi: 10.1371/journal.pntd.0010763 (PMC9499217; doi:10.1371/journal.pntd.0010763)

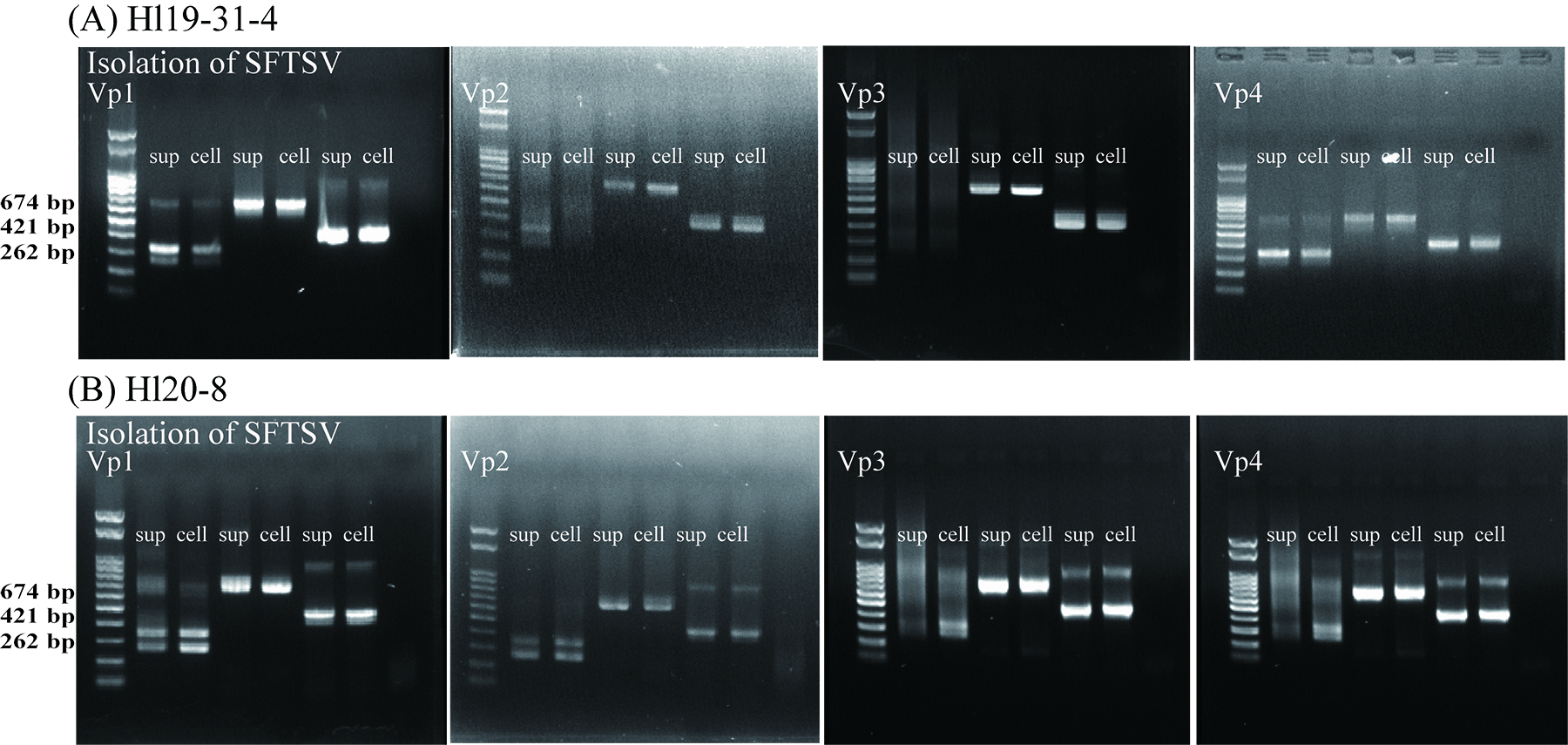

Supplement: S1 Fig — RT-PCR was continually performed to confirm isolation of SFTSV (A) Hl19-31-4 and (B) Hl20-8 strains from supernatants and cells from infected Vero E6 cells. (TIF) [file pntd.0010763.s001.tif]

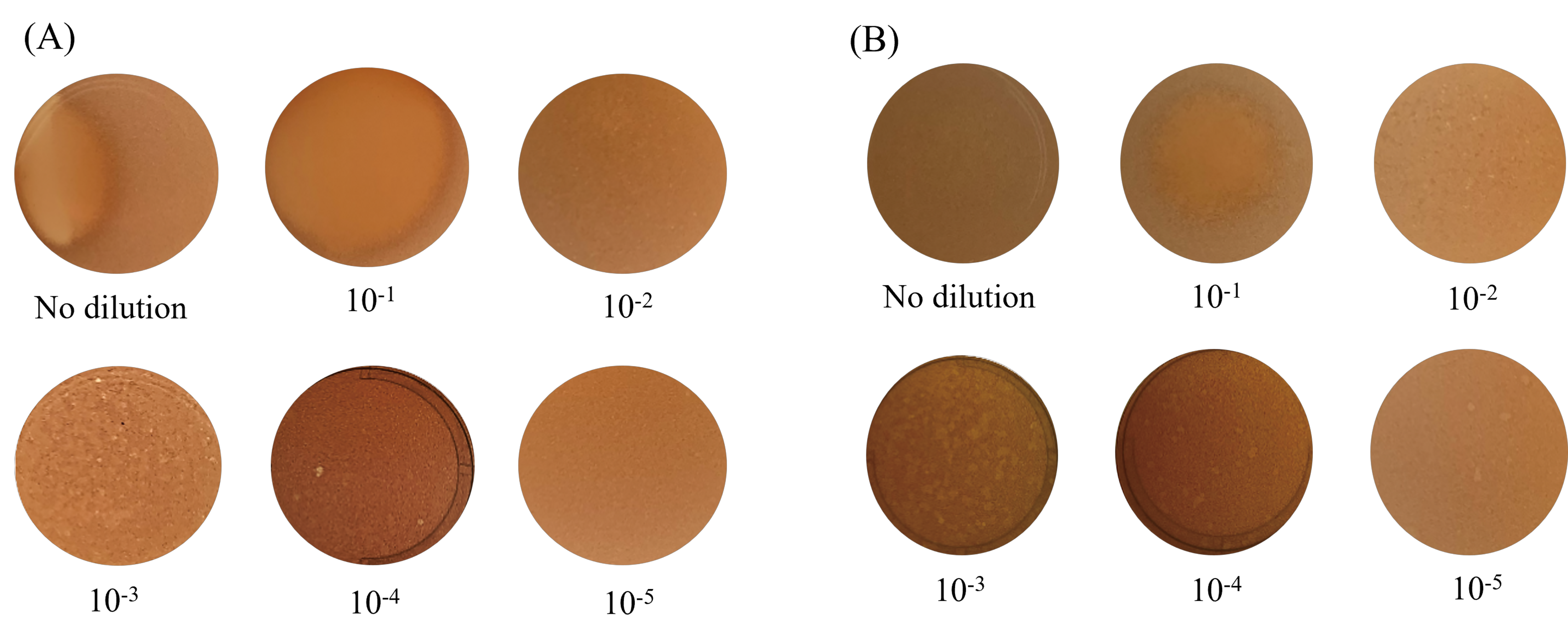

Supplement: S2 Fig — SFTSV Hl19-31-4 and Hl20-8 strains were quantified on 6-well plates with Vero E6 monolayers. Each well indicates different dilutions from top-left to right: no dilution, dilutions at 1:101, and 1:102, and bottom-left to right: dilutions at 1:103, 1:104, and 1:105, respectively. The first plaques of SFTSV (A) Hl19-31-4 and (B) Hl20-8 were confirmed at 5 days post-inoculation, and the number of infectious particles was 1.2×10^6^ and 2.0×10^6^ PFU/mL, respectively. (TIF) [file pntd.0010763.s002.tif]
