# Supplementary material for: Whole-genome sequencing and genetic diversity of severe fever with thrombocytopenia syndrome virus using multiplex PCR-based nanopore sequencing, Republic of Korea
Source: PLoS Negl Trop Dis. 2022 Sep 12;16(9):e0010763. doi: 10.1371/journal.pntd.0010763 (PMC9499217; doi:10.1371/journal.pntd.0010763)
Supplement: S1 Table — (PDF) [file pntd.0010763.s003.pdf]

S1 Table. Multiplex polymerase chain reaction primer information for severe fever with thrombocytopenia syndrome virus L, M, and S segments.

Segment	Primer	Sequence (5' → 3')	Position
L	SFTL01F	ACACAGAGACGCCCAGATGA	1–20
	SFTL02F	GACAGGCCAGGGCTGCCAGA	101–120
	SFTL03F	CAATCAATGCTGGGTTGATC	201–220
	SFTL04F	CTCAGAGGTATTCCCCATTA	301–320
	SFTL05F	ACCACTAGGAGCCATAACAT	401–420
	SFTL06F	ACCCGAGGGTCTTCTTTGGC	501–520
	SFTL07F	CTGCATAGCCAATGAGATCT	601–620
	SFTL08F	TCATTCTTCAGTTTGTTTGA	701–720
	SFTL09F	ATGTTGACTTCATCACCAAG	801–820
	SFTL10F	GCGGCTAGAGATCAATAGAT	901–920
	SFTL11F	ACAGTCAAACTCCCGCCCTG	1,001–1,020
	SFTL12F	GTGAGCTGTGGGCAAAGTGC	1,101–1,120
	SFTL13F	TCCAGAAAAAGATACCCCCA	1,201–1,220
	SFTL14F	GAGGGGAAGAAGTGGAAGAG	1,301–1,320
	SFTL15F	TCCTAACATTCAACAATCTT	1,401–1,420
	SFTL16F	CACAGAGCTCACTCATGCCC	1,501–1,520
	SFTL17F	GCTAGTGCTCTGAAGCAGCA	1,601–1,620
	SFTL18F	CCCATATCTTCTACAGCTTA	1,701–1,720
	SFTL19F	AACAGAGTTCAAAAGCCTGA	1,801–1,820
	SFTL20F	GTGGCTCCATGGCTGGTTCG	1,901–1,920
	SFTL21F	TCATCACCTTGACAAGGTAC	2,001–2,020
	SFTL22F	AACTAAGCTACAGGTATACC	2,101–2,120
	SFTL23F	TGGGGAGGAACATTCCATGC	2,201–2,220
	SFTL24F	CAGAGCTAAATGCCCTCGGA	2,301–2,320
	SFTL25F	CCCTAAGAAGCATGAATTCT	2,401–2,420
	SFTL26F	CTTGAGGAGCGTGTCCTGAG	2,501–2,520
	SFTL27F	CAGAAGTCCAGACCAAAGAG	2,601–2,620
	SFTL28F	GGCATGGAAGGCAGTTCTAG	2,701–2,720
	SFTL29F	GCCCGGCTCGTGCAGTTTGG	2,801–2,820
	SFTL30F	TAGAGAACCATGGGCTGAAG	2,901–2,920
	SFTL31F	AACAAAGCTAGCTCTAGTTC	3,001–3,020
	SFTL32F	GACCTAAGGTTTTTAGCTCA	3,101–3,120
	SFTL33F	TCTCATGGATGGACAAAGGG	3,201–3,220
	SFTL34F	GAGCTTTTACAAGTCTTATT	3,301–3,320
	SFTL35F	GCGATCATGATCAGCATACG	3,401–3,420
	SFTL36F	TGTTTGGGATTTATTCATCA	3,501–3,520
	SFTL37F	ACTGAGATGGATAGCAGCGT	3,601–3,620
	SFTL38F	GGGGCCTCATTCTCTCTTAC	3,701–3,720

	SFTL39F	TGGGGATGCTGATATCAGAC	3,801–3,820
	SFTL40F	AGCCTGCAAGACTACAGACC	3,901–3,920
	SFTL41F	ACATCGGGAGGAACTCTCAG	4,001–4,020
	SFTL42F	TGGAGCAGATAGATGAGAAT	4,101–4,120
	SFTL43F	GACTAGCAGCCTGAGTAAAG	4,201–4,220
	SFTL44F	GGCAGGGGCTCAACACAGAA	4,301–4,320
	SFTL45F	GAATGCTCTTCCCTCAGGCT	4,401–4,420
	SFTL46F	TGTCAGGAGCCGCATAGACT	4,501–4,520
	SFTL47F	CTTGGACCCCGTCTCCTCAA	4,601–4,620
	SFTL48F	TTAGCCATGTGCAGTTTAGG	4,701–4,720
	SFTL49F	CACCATAAGCCAAGTAGTCA	4,801–4,820
	SFTL50F	CTCAAGCATGTCTTGTTCAT	4,901–4,920
	SFTL51F	CATCAGAGGTCATCAGGAAA	5,001–5,020
	SFTL52F	ACAGTCAGGCACTCTAGGAG	5,101–5,120
	SFTL53F	ATGGAGGACACCCATGTTCA	5,201–5,220
	SFTL54F	TTGAATCCATCCGAAGGTTA	5,301–5,320
	SFTL55F	CAAGCCAGCATCAAGGACTG	5,401–5,420
	SFTL56F	AGGGGCGACATCCTCAACCT	5,501–5,520
	SFTL57F	CAGCATACCTCTGGAGCAAT	5,601–5,620
	SFTL58F	ACATGCCTCAGTTCTCCTGG	5,701–5,720
	SFTL59F	AAGCAAGGGCTGATGAGGTC	5,801–5,820
	SFTL60F	ACTTTTCAGATGTCATAGCT	5,901–5,920
	SFTL61F	GTTTGGAGAAGGAGTGGTGG	6,001–6,020
	SFTL62F	GAAGGCTGTAGAGGGCTCCT	6,101–6,120
	SFTL63F	TTTCTTGGGTGTCTGATCAG	6,201–6,220
	SFTL64F	GTGTCTGTGGGTGACTAGGG	6,301–6,320
	SFTL01R	ACACCTGTGGCATCGACAGT	150–131
	SFTL02R	GATCTTATATGCTTCAGAGA	250–231
	SFTL03R	CAGGGGTCATCCCATCAGAA	350–331
	SFTL04R	TTTGTCCTATATGCTGCCTC	450–431
	SFTL05R	CAGAACCCCTCCTGACGAGA	550–531
	SFTL06R	TGTCTGCATCCATAGATCTA	650–631
	SFTL07R	AATGTTCCTTCCACTCTTTC	750–731
	SFTL08R	ATAGGCCTCCACCTCTTTTG	850–831
	SFTL09R	GGAGGTCCTGAGTTTTGTCA	950–931
	SFTL10R	TCTATTGACTCACTTGATGG	1,050–1,031
	SFTL11R	CTCCACATTGCCCAGCGAGA	1,150–1,131
	SFTL12R	TGAATCGCCTATAGGTTATC	1,250–1,231
	SFTL13R	TCCTTCTCTTTCTTCTGGTT	1,350–1,331
	SFTL14R	GGACTCATCTCCATACCTCG	1,450–1,431
	SFTL15R	TGCTTAGGTTCCTCTTGAAT	1,550–1,531
	SFTL16R	TTCTTGATGATGAACTCACC	1,650–1,631

	SFTL17R	CCTCCTTGTCACGTCAGCCT	1,750–1,731
	SFTL18R	AGTTGACAAGATTCGTGAGC	1,850–1,831
	SFTL19R	ACCTGCTCTCTGAGCTCTGA	1,950–1,931
	SFTL20R	GGGAGGAGAGACAAAGCCCT	2,050–2,031
	SFTL21R	GCACCATGCAATCCAGATGC	2,150–2,131
	SFTL22R	TTCTCAAGGTTTGTGGACCG	2,250–2,231
	SFTL23R	TTCCATCTCCACAATCTTCT	2,350–2,331
	SFTL24R	ATGAGCAAGCAGCTCTGAGG	2,450–2,431
	SFTL25R	AGGTCCAGGATGTTCTTGGT	2,550–2,531
	SFTL26R	CATCTTCTCCAGCAGTTTGG	2,650–2,631
	SFTL27R	TGAAGAGACAGATTCGCATG	2,750–2,731
	SFTL28R	TCACAGACACACCTAGCCAT	2,850–2,831
	SFTL29R	TATAGAGCCAGGGCCAAGAC	2,950–2,931
	SFTL30R	TGTGGAATTTGGCTGGCATG	3,050–3,031
	SFTL31R	GATGACCTAGACTCAGATTT	3,150–3,131
	SFTL32R	CATTCCTGTCTCTGTCTTTA	3,250–3,231
	SFTL33R	CCATGTAGCCCTCCTTGAGC	3,350–3,331
	SFTL34R	GATCGGACTTCATCCATGTC	3,450–3,431
	SFTL35R	ACAATACACTGTGTTCACTG	3,550–3,531
	SFTL36R	CAAGGGCTTCAGTCTCTGAG	3,650–3,631
	SFTL37R	TGCAGGAGCTGAGCGCACTG	3,750–3,731
	SFTL38R	CATGAGGAAGAACCCTAGGG	3,850–3,831
	SFTL39R	CATTAAAATAATATGCATAC	3,950–3,931
	SFTL40R	CTGTCCCCCCAGTACACCAT	4,050–4,031
	SFTL41R	GTTGGCAGCTCTCCTGTAAA	4,150–4,131
	SFTL42R	CTGCTGCCACCACCCGAGGC	4,250–4,231
	SFTL43R	GACATCATCAGCAGTTTTAT	4,350–4,331
	SFTL44R	AAGCAATGTGCATACTCTGT	4,450–4,431
	SFTL45R	TGCACCGCAAGTCCACTGGC	4,550–4,531
	SFTL46R	AATGAGGCCCTAAGTTTGTC	4,650–4,631
	SFTL47R	TGATTTGGCATCAACGTGGG	4,750–4,731
	SFTL48R	CTAGGCTAAAACCAGGGAAG	4,850–4,831
	SFTL49R	TACTCCTCAGTGTATGGGCC	4,950–4,931
	SFTL50R	CGATAAGAGGCATAAAGTCA	5,050–5,031
	SFTL51R	TAATGAATGTCTTCTGGGGC	5,150–5,131
	SFTL52R	TTACTAGTCCCATCTCCATC	5,250–5,231
	SFTL53R	CACCCTGTTGTTGATCCCAA	5,350–5,331
	SFTL54R	CCCTTTCCATAATCCTGACT	5,450–5,431
	SFTL55R	ATGACCCTTCCTTCTTGTAT	5,550–5,531
	SFTL56R	CTCCTTCTTCCCAAAGGAGA	5,650–5,631
	SFTL57R	TGTCAATGCCTTGGGTCTTC	5,750–5,731
	SFTL58R	ACATTCTTCTCCACCATCTC	5,850–5,831

	SFTL59R	AATGTCAAGTGATCCCTCTG	5,950–5,931
	SFTL60R	TCTTCCACCTCAGCAGACCA	6,050–6,031
	SFTL61R	CTTACCCATGATCGCACACA	6,150–6,131
	SFTL62R	TGCTGTCCTCAGGAATTTGC	6,250–6,231
	SFTL63R	ATCAACTGCGTGCCAACCAT	6,350–6,331
	SFTL64R	ACACAAAGACCGCCCAGATC	6,368–6,349
M	SFTM01F	ACACAGAGACGGCCAACAAT	1–25
	SFTM02F	GACCCATCCACTCAAACAAG	101–120
	SFTM03F	CAGAAACCACTCACAATTTC	201–220
	SFTM04F	CTTCTAAGCCCCTGTGATGC	301–320
	SFTM05F	TCTTTTTTGAACGGCCAACT	401–420
	SFTM06F	GACTAGTTCTGGTCTTCTGC	501–520
	SFTM07F	AAGAGTTTTAGCCAAAGTGA	601–620
	SFTM08F	CATGGATGGATGTTGGCCAC	701–720
	SFTM09F	AGGGACTGGGCCTTGTTCTG	801–820
	SFTM10F	GGGGAAGAGGCATCTGAAGC	901–920
	SFTM11F	ATGGTTTCTCCAGAATGATG	1,001–1,020
	SFTM12F	GAGGCTAATCACTCTAACCA	1,101–1,120
	SFTM13F	TTCCACTCAGGATCATTGGT	1,201–1,220
	SFTM14F	ATGGTTGTGATGCAGTGGAC	1,301–1,320
	SFTM15F	CCTTGGATATGCAGGCCTCA	1,401–1,420
	SFTM16F	ATAAAGAAACTAATGAGAAC	1,501–1,520
	SFTM17F	ACCAAGATGATGTTAGGATT	1,601–1,620
	SFTM18F	GGGCTGTGATGAGATGGTCC	1,701–1,720
	SFTM19F	CCTGCGGTGAACCCAGGACA	1,801–1,820
	SFTM20F	AATGTAAGAAGTCATCATCA	1,901–1,920
	SFTM21F	CCCTCATTTCACATCCAACT	2,001–2,020
	SFTM22F	GCCTGCGGCTGCTTTAATGC	2,101–2,120
	SFTM23F	GGGTCCCATCAGCAGTCATA	2,201–2,220
	SFTM24F	TGTGACTTACTTGGGCTCAG	2,301–2,320
	SFTM25F	CAGGCTGGCATGGGGGTTGT	2,401–2,420
	SFTM26F	ACCTTGTGGGCATAGAGCTA	2,501–2,520
	SFTM27F	TGGGGGGCTGAGGTTTGAGA	2,601–2,620
	SFTM28F	AGAGGCCTTCGACTGAGCCT	2,701–2,720
	SFTM29F	CCATCAAACTGCATAGCAGC	2,801–2,820
	SFTM30F	CAGTCTCAGTTTTGACCATG	2,901–2,920
	SFTM31F	GATGTCCCAAAATTTGTGGA	3,001–3,020
	SFTM32F	TGTTTGGCAATGGGCTGAGT	3,101–3,120
	SFTM33F	AACAAAACAGGTATTTCGAT	3,201–3,220
	SFTM34F	CAGTGTGCATGTTTCGTGGT	3,301–3,320
	SFTM01R	GTAACCAAGCAGGTGGGGTA	150–131
	SFTM02R	AGCGTCCACCTCGCTGCCCT	250–231

	SFTM03R	TTCTTCACGACAAGCATCCC	350–331
	SFTM04R	GCCACAGCCCCAGCCTACGT	450–431
	SFTM05R	CATCACCTATCCAGAGAACC	550–531
	SFTM06R	ATGCCATCAATTTTGCATAT	650–631
	SFTM07R	CTTGTGCTCCCTCATGATGA	750–731
	SFTM08R	CACTGGTCTTGCAAGTCTTT	850–831
	SFTM09R	GGCTTGTGCACAAGTGAGCA	950–931
	SFTM10R	TTGCTCTGGTTGGTTCACCT	1,050–1,031
	SFTM11R	AAGCACAGACAGTAGCTGAC	1,150–1,131
	SFTM12R	GCCCCTTTGACGTGTATTGC	1,250–1,231
	SFTM13R	ATTTTTTAGAAACTCACGAC	1,350–1,331
	SFTM14R	CAATTGCCTTAAGGACATTG	1,450–1,431
	SFTM15R	TCCATCAATTTCCCCATCAA	1,550–1,531
	SFTM16R	ATGCCTTACCCTTCTGGGTC	1,650–1,631
	SFTM17R	GCCTGCACGAAACAAGTTTA	1,750–1,731
	SFTM18R	CCAGGTGCCGTGAAGTGCAG	1,850–1,831
	SFTM19R	ACACCTGGACCGGGCATCAG	1,950–1,931
	SFTM20R	CCATCTTACCTGCCCAATCA	2,050–2,031
	SFTM21R	CATTTCCTCCAGAAGATGCA	2,150–2,131
	SFTM22R	CCTCACCTCCCCTGAGGGCA	2,250–2,231
	SFTM23R	ACAGGTCAGTCAAGCCAGAC	2,350–2,331
	SFTM24R	CTACTGCACTGTATCTCTCC	2,450–2,431
	SFTM25R	CTTAGAGTAGCACACAGCGT	2,550–2,531
	SFTM26R	CAGATATTTTACCCTGGCTG	2,650–2,631
	SFTM27R	TCCCCTGTGCAAGTAGCAGT	2,750–2,731
	SFTM28R	TTTGCATCTTACATGGGCAG	2,850–2,831
	SFTM29R	AGTTCAGTTGGCACTGCTCA	2,950–2,931
	SFTM30R	GTACTATGATATGTCTGCAT	3,050–3,031
	SFTM31R	TAGAACCCCTATCACCCCCA	3,150–3,131
	SFTM32R	ATTTGCCCATCTAAGCCAGC	3,250–3,231
	SFTM33R	AACCTCCATGCTCTAAGAAT	3,350–3,331
	SFTM34R	ACACAAAGACCGGCCAACAC	3,450–3,431
S	SFTS01F	ACACAAAGAACCCCCTTCAT	1–20
	SFTS02F	GCCATCTCTAGGAGAGTACC	101–120
	SFTS03F	AACACAGTATGGTTGTTTGG	201–220
	SFTS04F	ACTCCCAGAGAGAGCTGAGT	301–320
	SFTS05F	GGCAATGGAAACTGGGAGAG	401–420
	SFTS06F	GAAGCCATAGAGAAGCGAAG	501–520
	SFTS07F	TTGCAAGGCTAAGAGGCATG	601–620
	SFTS08F	CAAAAAGAAGACAGATGGCT	701–720
	SFTS09F	TGCCCATTCCACAACCTCAT	801–820
	SFTS10F	AGGAGGTCTAATTTTGTCGA	901–920

	SFTS11F	CAGCTCTGCTGGGGACCCCA	1,001–1,020
	SFTS12F	GAGAGGATCCCTGAAGGAGT	1,101–1,120
	SFTS13F	TCCATCAGGGTCTTGGTCGT	1,201–1,220
	SFTS14F	GGTTCATGACGGCTGGCCCC	1,301–1,320
	SFTS15F	TGAGAGCCTGGTCTCTGCCC	1,401–1,420
	SFTS16F	ATCTTATTGCCTCGAGTCAG	1,501–1,520
	SFTS17F	GATCAAGGCCTTCATAGGCT	1,601–1,620
	SFTS01R	AGCATTCAACGAGGTCCCTC	150–131
	SFTS02R	TCAAGCTGGTGAAGAGGATT	250–231
	SFTS03R	AGATGGCTTCCCACTAGGCC	350–331
	SFTS04R	TTATCCTGTGGAAGAGGCCC	450–431
	SFTS05R	ATTAGGATATCAGGTAACCC	550–531
	SFTS06R	GGAAGAAGTGAACAAGTGGT	650–631
	SFTS07R	GCTCATCATCCTCATCCAAG	750–731
	SFTS08R	GCTGCAGTTCTCAGCTCCTC	850–831
	SFTS09R	ATCCAATTAGGCTAAATTTC	950–931
	SFTS10R	GCTGAAGGCCAAGGGAATCC	1,050–1,031
	SFTS11R	TGCGCGGAGCCAGCAAGACA	1,150–1,131
	SFTS12R	CTGATTCCAACTGCAGGGGT	1,250–1,231
	SFTS13R	AGCAGCAGCCTTAAAGGAGT	1,350–1,331
	SFTS14R	CACTCCAGGAGAAATATGGG	1,450–1,431
	SFTS15R	AAGGATACTAAGTTCATCAT	1,550–1,531
	SFTS16R	GACTGAGCTTGAGGATTTCG	1,650–1,631
	SFTS17R	ACACAAAGAACCCCCAAAAA	1,746–1,727

3
