# Supplementary material for: Whole-genome sequencing and genetic diversity of severe fever with thrombocytopenia syndrome virus using multiplex PCR-based nanopore sequencing, Republic of Korea
Source: PLoS Negl Trop Dis. 2022 Sep 12;16(9):e0010763. doi: 10.1371/journal.pntd.0010763 (PMC9499217; doi:10.1371/journal.pntd.0010763)
Supplement: S2 Table — (PDF) [file pntd.0010763.s004.pdf]

S2 Table. Oligonucleotide primers to obtain 3'and 5'ends genomic sequences of L, M and S segments of severe fever with thrombocytopenia syndrome virus using rapid amplification of cDNA ends polymerase chain reaction in this study.

Segment	Primer	Sequence (5' → 3')	Position
L	SFTSL 5END 1st	ACCTGTACATGAGCTCCTCT	599-580
	SFTSL 5END 2nd 1	CTCCATGATATCAACACGCC	499-480
	SFTSL 5END 2nd 2	CTTGAGATTGGGTCCCTATA	480-461
	SFTSL 5END 2nd 3	GTGGTTGAAAATTCAACCAC	402-383
	SFTSL 3END 1st	ACCTATCTTGTGGTCTGCTG	5,974-5,993
	SFTSL 3END 2nd 1	TGGTGGCTGTGAGCTATT	6,018-6,035
	SFTSL 3END 2nd 2	AAGCTGCCATCACAATGTGT	6,069-6,088
	SFTSL 3END 2nd 3	AAGCTGCCATCACAATGTGT	6,165-6,184
M	SFTSM 5END 1st	CCTTGTAGCACACAAAATCC	799-780
	SFTSM 5END 2nd 1	GCAACATCACCTATCCAGAG	554-535
	SFTSM 5END 2nd 2	TTGTGCTCCCTCATGATG	749-732
	SFTSM 5END 2nd 3	AGGCAACATCACCTATCC	556-539
	SFTSM 3END 1st	GGTAAAATATCTGGTAGCCC	2,638-2,657
	SFTSM 3END 2nd 1	CGCCAAAGTCTCCATCAAA	2,790-2,808
	SFTSM 3END 2nd 2	AGATGCAAAGGGGATGAGAC	2,842-2,861
	SFTSM 3END 2nd 3	ATCATAGTTCTGTGCCCACA	3,041-3,060
S	SFTSS 5END 1st	AGTGTGCCATCAGATATGG	789-771
	SFTSS 5END 2nd 1	ATGAGCCATCTGTCTTCTT	723-705
	SFTSS 5END 2nd 2	AAGAAGTCCCAACAGTCTA	688-670
	SFTSS 5END 2nd 3	GTGGTGGTTCAAGCTCAT	635-618
	SFTSS 3END 1st	CCACTTCACCCGAACATCAT	1,050-1,069
	SFTSS 3END 2nd 1	CCCTGAAGGAGTTGTAAACC	1,109-1,128
	SFTSS 3END 2nd 2	CGCATCTTCACATTGATAGT	1,147-1,166
	SFTSS 3END 2nd 3	AAGGCCATGCACATCATCTC	1,258-1,277
