# Supplementary material for: Whole-genome sequencing and genetic diversity of severe fever with thrombocytopenia syndrome virus using multiplex PCR-based nanopore sequencing, Republic of Korea
Source: PLoS Negl Trop Dis. 2022 Sep 12;16(9):e0010763. doi: 10.1371/journal.pntd.0010763 (PMC9499217; doi:10.1371/journal.pntd.0010763)
Supplement: S3 Table — (PDF) [file pntd.0010763.s005.pdf]

S3 Table. Summary of mapped reads and average depth of multiplex polymerase chain reaction-based nanopore sequencing of severe fever with thrombocytopenia syndrome virus.

Sample	Ct value	Running time	Total reads	Reads mapped / Total reads (%)	L segment			M segment			S segment		
					Reads mapped	Depth of coverage	Genome coverage (%)	Reads mapped	Depth of coverage	Genome coverage (%)	Reads mapped	Depth of coverage	Genome coverage (%)
Hl19-31-4	22.1	5 min	2,958	97.87	677	25.01	99.43	867	50.08	99.47	1,351	196.52	99.20
		15 min	7,877	97.68	1,810	65.65	99.43	2,293	129.82	99.47	3,591	521.64	99.20
		30 min	16,729	97.63	3,806	139.70	99.43	4,736	269.86	99.47	7,790	1,145.35	99.20
		1 h	33,391	97.64	7,629	284.03	99.43	9,322	537.99	99.47	15,651	2,324.37	99.20
		3 h	100,926	97.32	23,167	877.74	99.43	27,236	1,598.43	99.47	47,818	7,247.77	99.20
		6 h	189,105	96.96	43,221	1,665.73	99.43	49,799	2,955.82	99.47	90,336	13,924.35	99.20
Hl19-31-13	25.1	5 min	991	97.78	354	13.26	99.43	296	21.74	99.47	319	55.14	99.20
		15 min	1,982	96.97	703	27.07	99.43	588	43.44	99.47	631	109.20	99.20
		30 min	5,935	97.46	2,121	81.95	99.43	1,785	135.30	99.47	1,878	327.75	99.20
		1 h	11,832	97.48	4,299	168.61	99.43	3,486	262.88	99.47	3,749	652.71	99.20
		3 h	36,346	97.04	13,069	523.57	99.43	10,534	814.14	99.47	11,667	2,097.02	99.20
		6 h	70,405	96.53	24,913	1,014.77	99.43	20,054	1,569.32	99.47	22,992	4,223.45	99.20
Hl20-8	20.8	5 min	2,878	97.56	1,074	39.72	99.43	452	28.14	99.47	1,352	206.07	99.20
		15 min	6,898	97.78	2,558	95.24	99.43	1,100	69.63	99.47	3,087	471.68	99.20
		30 min	14,428	97.64	5,491	205.93	99.43	2,351	148.39	99.47	6,586	1,016.79	99.20
		1 h	29,499	97.55	11,013	418.47	99.43	4,657	293.59	99.47	13,105	2,043.57	99.20
		3 h	88,290	97.32	32,848	1,275.23	99.43	13,408	871.44	99.47	39,668	6,330.94	99.20
		6 h	165,103	97.06	60,917	2,404.57	99.43	24,240	1,601.13	99.47	75,092	12,234.78	99.20
