# Supplementary material for: Whole-genome sequencing and genetic diversity of severe fever with thrombocytopenia syndrome virus using multiplex PCR-based nanopore sequencing, Republic of Korea
Source: PLoS Negl Trop Dis. 2022 Sep 12;16(9):e0010763. doi: 10.1371/journal.pntd.0010763 (PMC9499217; doi:10.1371/journal.pntd.0010763)
Supplement: S4 Table — (PDF) [file pntd.0010763.s006.pdf]

S4 Table. Mapped reads and average depth of multiplex polymerase chain reaction-based Illumina sequencing of severe fever with thrombocytopenia syndrome virus.

Sample	Total reads	Reads mapped / Total reads (%)	L segment			M segment			S segment		
			Reads mapped	Depth of coverage	Genome coverage (%)	Reads mapped	Depth of coverage	Genome coverage (%)	Reads mapped	Depth of coverage	Genome coverage (%)
Hl19-31-4	685,876	98.1	303,117	5,762.70	99.43	243,482	8,741.18	99.47	126,072	8,747.15	99.20
Hl19-31-13	950,480	98.7	384,735	7,336.03	99.43	368,157	13,232.43	99.47	185,580	12,879.78	99.20
Hl20-8	4,140,773	96.8	745,327	14,373.88	99.43	1,464,272	51,928.13	99.47	1,799,842	124,393.79	99.20
