# Supplementary material for: Whole-genome sequencing and genetic diversity of severe fever with thrombocytopenia syndrome virus using multiplex PCR-based nanopore sequencing, Republic of Korea
Source: PLoS Negl Trop Dis. 2022 Sep 12;16(9):e0010763. doi: 10.1371/journal.pntd.0010763 (PMC9499217; doi:10.1371/journal.pntd.0010763)
Supplement: S5 Table — (PDF) [file pntd.0010763.s007.pdf]

S5 Table. Accession numbers of genomic sequences of severe fever with thrombocytopenia syndrome virus used in this study.

Sample	Accession number		
	L segment	M segment	S segment
**Hl19-31-4/MiSeq**	**OM160952**	**OM160953**	**OM160954**
**Hl19-31-4/MinION**	**OM160961**	**OM160962**	**OM160963**
**Hl19-31-13/MiSeq**	**OM160955**	**OM160956**	**OM160957**
**Hl19-31-13/MinION**	**OM160979**	**OM160980**	**OM160981**
**Hl20-8/MiSeq**	**OM160958**	**OM160959**	**OM160960**
**Hl20-8/MinION**	**OM160982**	**OM160983**	**OM160984**
15KS67	MG737038	MG737147	MG737255
15KS75	MG921169	MG921170	MG921171
15MS32	MG737019	MG737128	MG737236
16KS100	MG737067	MG737176	MG737284
16KS104	MG737074	MG737183	MG737291
16KS112	MG737076	MG737185	MG737293
16KS126	MG737079	MG737188	MG737296
16KS45	MF094749	MF094774	MF094799
16KS71	MG737051	MG737160	MG737268
16KS80	MG737054	MG737163	MG737271
16MS239	MG737047	MG737156	MG737264
16MS286	MG737052	MG737161	MG737269
16MS315	MG737064	MG737173	MG737281
16MS344	MG737069	MG737178	MG737286
AFMC17-1	MH375689	MH375688	MH375687
AHL/China/2011	JQ670934	JQ670930	JQ670932
CB1	KY789433	KY789436	KY789439
CB3	KY789435	KY789438	KY789441
Jilin	KT890280	KT890281	KT890282
JNU-1	LC516196	LC516197	LC516198
JS2014-39	KR230764	KR230784	KR230804
JS2014-*H. longicornis*-01	KR230765	KR230785	KR230805
JS2014-*H. longicornis*-02	KR230766	KR230786	KR230806
JS2014-*H. longicornis*-03	KR230767	KR230787	KR230807
JS4/China/2010	HQ141604	HQ141605	HQ141606
KACNH3	KP663743	KP663744	KP663745
KADGH	KU507543	KU507548	KU507553
KADGH4	KU507544	KU507549	KU507554
KAGBH5	KP663737	KP663738	KP663739
KAGBH6	KP663740	KP663741	KP663742

KAGNH	KU507545	KU507550	KU507555
KAGNH4	KU507546	KU507551	KU507556
KAGWH2	MG736986	MG737095	MG737203
KAGWH3	KP663734	KP663735	KP663736
KAGWT	KY273136	KY273137	KY273138
KAICH	MG736981	MG737090	MG737198
KAJJH	MG736973	MG737082	MG737191
KAJNH2	KU507547	KU507552	KU507557
KASJH	KP663731	KP663732	KP663733
KASUH	MG736992	MG737101	MG737209
KAUSH	MG736984	MG737093	MG737201
NB32/CHN/2013	KR698352	KR698339	KR698326
NB38/CHN/2013	KR698354	KR698341	KR698328
SD4/China/2010	HM802202	HM802203	HM802204
SDLZtick12/2010	JQ684871	JQ6A84872	JQ684873
SPL005A	AB817982	AB817990	AB817998
SPL035A	AB817986	AB817994	AB818002
SPL087A	AB983515	AB985309	AB985541
USAMRIID HLP23 Tick	MN395043	MN395044	MN395045
YG1	AB817979	AB817987	AB817995

Newly recovered SFTSV genomes in this study are shown in bold font.
